# Dynamic capabilities of public sector organisations: A case study of forest and land fires in Indonesia

**DOI:** 10.4102/jamba.v18i1.2056

**Published:** 2026-08-21

**Authors:** Geovani Meiwanda, Agus Pramusinto, Bevaola Kusumasari, Novi Paramita Dewi

**Affiliations:** 1 Department of Public Administration, Faculty of Social and Political Sciences, Universitas Riau, Pekanbaru, Indonesia; 2 Center of Peatland and Disaster Studies (CPDS), Universitas Riau, Pekanbaru, Indonesia; 3 Department of Public Policy and Management, Faculty of Social and Political Science, Universitas Gadjah Mada, Yogyakarta, Indonesia

**Keywords:** disaster decentralisation, disaster management, local government, dynamic capabilities, forest and land fire disaster, peatland fire

## Abstract

This article presents findings from a longitudinal study on decentralisation in disaster management. The 2015 regional disasters exposed a fundamental incapacity in local crisis management, compelling the central government to reassume control of a system that had been formally decentralised since 2007. When fires recurred in 2019, the regional government had developed partial institutional responses, yet remained unable to fully initiate an independent crisis response, revealing that decentralisation had transferred authority without transferring capability. This study applies Teece’s dynamic capabilities framework, operationalised through the pillars of sensing, seizing, and transforming, to explain why this gap persists. Data were collected through in-depth interviews with key actors during the crisis and post-disaster periods, triangulated with official government archives and local media documentation. The findings offer three theoretical contributions that extend Teece’s framework for public-sector applications. Firstly, a learning phase is identified as a necessary precondition for dynamic capabilities to form in organisationally immature, decentralised systems. Secondly, an asymmetric-substitutional pattern is observed, in which macro-level policy advances consistently outpace operational capacity at the field level. Thirdly, the study introduces networked dynamic capability.

**Contribution:**

This study describes the process or evolution that resulted in public organisations specifically disaster management, using a decentralised system. Disaster decentralisation cannot occur immediately if the public organisation is not prepared, and transformation only occurs after a crisis occurs.

## Introduction

Discussing disasters today is no longer confined to a limited context, as disasters do not exist, but hazards do. In a way, disasters are a societal failure. Therefore, what is needed is a shared perspective from a disaster-prone country to continuously recalibrate, as dynamic conditions require more developed responses. The dynamic nature of disasters necessitates risk-based assessments and adaptation to the evolving nature of destructive phenomena over time (Izaddoost & Heydari 2017). Past experiences are the most appropriate basis for building risk-based learning to face future disasters (De Vries 2019).

Dynamic capabilities (DC), characterised by resources that continuously adapt and develop, are an important part of disaster management (De Andrade et al. 2021). This is filled out and integrated with development plans that account for the impact of disasters and ensure clear technical coordination (Pradhan & Xu 2018). Coordination within the bureaucracy for disaster affairs involves all elements of government, including crisis management approaches, leadership support, coordination patterns and organisational culture (Carayannopoulos 2017). Given the impact of disasters, disaster management costs are integral to an effective disaster management strategy (Shahmohammadian et al. 2025). Thus, disaster management is not only about resources but also about an organisation that integrates coordination capabilities across bureaucratic elements and is realistic in considering the impact of the disaster itself, forming a single adaptive strategic identity.

The success of government institutions in disaster resilience is supported by two factors: Learning and experience. Experience with past disaster crises can improve responses to future disasters (Haraty & Utaberta 2019). This relates to the dynamics of decision-making and collaboration patterns in disaster management (Arain 2015). Inclusive elements that make the community part of the government’s supporters in pre-disaster, disaster and post-disaster conditions (Sandaran & Selvaraj 2021). Accumulated experience strengthens decision-making capacity, making it increasingly relevant when supported by external factors integral to the disaster management system.

The government’s implementation of disaster management needs to be guided by strategic and inclusive policies (Djalante 2018; Prianto & Abdillah 2023). A growing body of studies criticises the poorly established disaster system; the centralisation of disaster management limits its effectiveness (Bang 2013), and this will later be related to poor accountability and transparency in government commitments and principles in disasters (Dania & Juwitasari 2025).

A crucial question is how government institutions ultimately develop adaptive and sustainable disaster management capabilities. Disaster management capabilities do not emerge spontaneously; they must be developed (Saadi 2023). The construction of disaster governance implemented by public organisations, when viewed through the lens of the DC concept, provides a visualisation of the evolutionary process that fosters adaptation. The DC, as introduced by Teece, also requires an organisation to understand its needs amid dynamic change and to survive in those conditions (Teece 1998). This is closely related to calibrating an organisation’s experience into learning, which is then successfully translated into transformation.

One of the disasters in Indonesia that remains a significant issue because of its high potential for recurrence is forest and land fires. Researchers at Center for International Forestry Research (CIFOR) Indonesia also stated that political issues, land management and the commitment of local government leadership are crucial factors in determining the cause of these disasters (Purnomo et al. 2017, 2021). This disaster occurred as a result of three conditions, namely deliberate fire (human), peat land and climate anomalies (Yunianto 2020). The economic and health impacts were felt not only by the Indonesian government and people but also by neighbouring countries such as Singapore and Malaysia. The resulting haze from the disaster drew attention from affected countries and led to criticism of the Indonesian government’s inability to manage this recurring issue (Carmenta et al. 2017; Meiwanda & Nizmi 2021).

The crucial issue regarding the forest and land fire disaster in Indonesia is whether responsibility lies entirely with the state or primarily with local governments. A resilient disaster management system requires more than just appropriate technology and policies; it also requires clear roles and responsibilities between the central and regional governments (Pamungkas et al. 2023; Retno Susilorini et al. 2021). When this does not go hand in hand, it can cause losses across various sectors (Tampubolon et al. 2018). In Indonesia, since 2007, a decentralised disaster management system has been in place, in which disaster management is not fully managed by the state but by regional governments, namely at the provincial or district level.

Riau Province is one of six provinces in Indonesia that experienced this disaster between 2015 and 2019 (Nurhidayah et al. 2023). During the 2015 disaster, it did not yet have the capability to control fire and smoke haze (Meiwanda 2016). There is an inability because of the failure to learn from past events (Helfat & Peteraf 2009). Because Riau Province experienced a similar incident in 1997, and from 2015 until 2019, fires occurred every year in several areas without any evaluation, disaster management was not a main focus in development plans or in regional government disaster organisations.

In accordance with the three causes of this disaster, firstly, is that humans are dominant because of deliberate actions and display normal land management conditions in Riau (Rochwulaningsih et al. 2022). Previous research shows that policies in several vulnerable areas drive deforestation rates in line with the widespread distribution of forest and land fires (Purnomo et al. 2019). The empirical evidence is that the 2015 disaster marked the beginning of a decentralised era in Indonesia, necessitating recentralisation because of local governments’ inability to cope with disasters. The dominant actors that emerged in 2015 was fully adopted in the face of the recurring fire disaster in 2019. The difference is that the Riau Provincial Government has experience in disaster coordination and emergency budgeting.

The current snapshot spans the period since 2015, during which the Riau Provincial Government has developed various adaptations and independent policies. The number of fires in 2025 is still 21 843 ha burned, while in the 2019 disaster, 90 550 ha burned. What is interesting is not the decline but the conclusion that the local government’s performance in disaster management is improving, even as hotspots are still found on corporate land. What capabilities will the Riau Provincial Government ultimately develop to ensure that the annual fires and current climate conditions do not cause a repeat disaster in Riau? The fires remain a hotly debated issue among researchers and Non-governmental Organisations (NGOs) working on environmental issues in Indonesia and internationally.

## Research methods and design

There are several key points before establishing the research method used in this article, namely defining research boundaries to avoid expanding the scope of bias for the author and readers. Conceptually, using the analytical tool of Teece’s concept, namely DC, which does not measure the performance resulting from the concept, but rather focuses on one argument that this concept describes the evolution of organisational adaptation. Therefore, this study discusses the DC concept in relation to forest and land fires in Riau. The intended unit of analysis is the Riau Provincial Government, a regional government with the capacity to manage disasters in accordance with Indonesia’s decentralised disaster system.

This study adopted a qualitative research design to capture the DC process established by the Riau Provincial Government. The primary data were constructed from longitudinal interviews with elite bureaucratic actors involved in the 2015 and 2019 disasters, as well as those currently in office. To ensure objectivity in summarising, analysing and presenting the contributions of this study, data collection was also supported by Focus Group Discussions (FGDs) involving various government elements in Riau Province, such as the regional development planning, forestry and environmental sectors, regional disaster management organisations, NGOs, community groups called MPAs and companies, with two samples in two different districts in Riau. The interviews and FGDs were synchronised with disaster archives from Indonesian print media documented by the National Library of Indonesia.

The data obtained were then organised through thematic coding in accordance with the operationalisation of the three pillars of DC: Sensing, seizing and transforming, thereby enabling triangulation to narrow the analysis of the research findings. The first stage was conducted through thematic analysis. This procedure is highly flexible, allowing for a variety of approaches, including inductive and deductive methods (Robinson 2016). Thematic analysis was applied to reduce, categorise and map data patterns from interviews and documentation studies. This study uses an inductive logic flow to capture the complexity of the adaptation process over time. The goal is not to prove mechanical cause-and-effect relationships, but rather to comprehensively explore meaning, understand how the pillars of dynamic capabilities are shaped by their ecosystems, and ultimately build a new theoretical synthesis firmly rooted in the empirical context (Creswell 2018). Qualitative Longitudinal Research (QLR) is a methodology that involves collecting qualitative data over an extended period to understand change and continuity in participants’ experiences and perspectives. This research explores how individuals interpret and respond to social change over time. Qualitative Longitudinal Research emphasises the passage of time and its impact on individual lives, making it suitable for studying dynamic processes and transitions (Rochwulaningsih et al. 2022). The research process using QLR involves participatory deliberation, notes and interviews (Rees & Ottrey 2026).

### Ethical considerations

Ethical clearance to conduct this study was obtained from the Universitas Gadjah Mada and The Ethics Research Committee (Ref. No. KE/UGM/215/EC/2025).

## Results

### Learning phase and configuration phase in the dynamic capabilities of the Riau provincial government

The DC concept does not distinguish between the learning and configuration phases. However, DC exists during reconfiguration, making it identical over time. Empirically, this study uses longitudinal data from two disaster periods in Riau to connect these theoretical claims with the initial condition in 2015, when Riau Province implemented disaster decentralisation without the necessary capabilities. On the other hand, organisational theory is generally highly developed because of the dynamic conditions within the bureaucratic realm (Bozeman & Moulton 2011). Resources and behaviour within an organisation reflect the responses of a public agency (Whitford et al. 2020).

The DC cannot immediately operate during a disaster because of immature decentralisation. This provides a new perspective and serves as a bridge to Teece’s argument when operationalising it in public organisations with immature decentralised systems. The learning phase does not indicate a lack of dynamic capabilities in disaster management; rather, it serves as a first step for local governments to independently develop their own dynamic capabilities. Understanding DC is a consciously constructed process within an organisation, one synonymous with knowledge and resilience. There are three pillars in the operationalisation of DC, and these pillars cannot directly describe the adaptations of local governments within the analysis unit of this article. The following is the operationalisation, contextualised in the context of forest and land fire disasters.

One of the supports for the literature study is that this concept is used in disaster management in developing countries to formulate strategies because of limited resources (Wided 2022). A public agency that understands disasters can interpret them as learning experiences and then use that experience as capital to form collaborations, because disasters are close and unpredictable events (Sinha & Ola 2021)

The current unpredictable crisis has been widely debunked, including at a forum hosted by the United Nations Office for Disaster Risk Reduction. The campaign, ‘Disasters are not natural’, advocated that disasters are caused by someone and have a significant impact. This is why the evolution of government responses to disasters is crucial.

**FIGURE 1:**
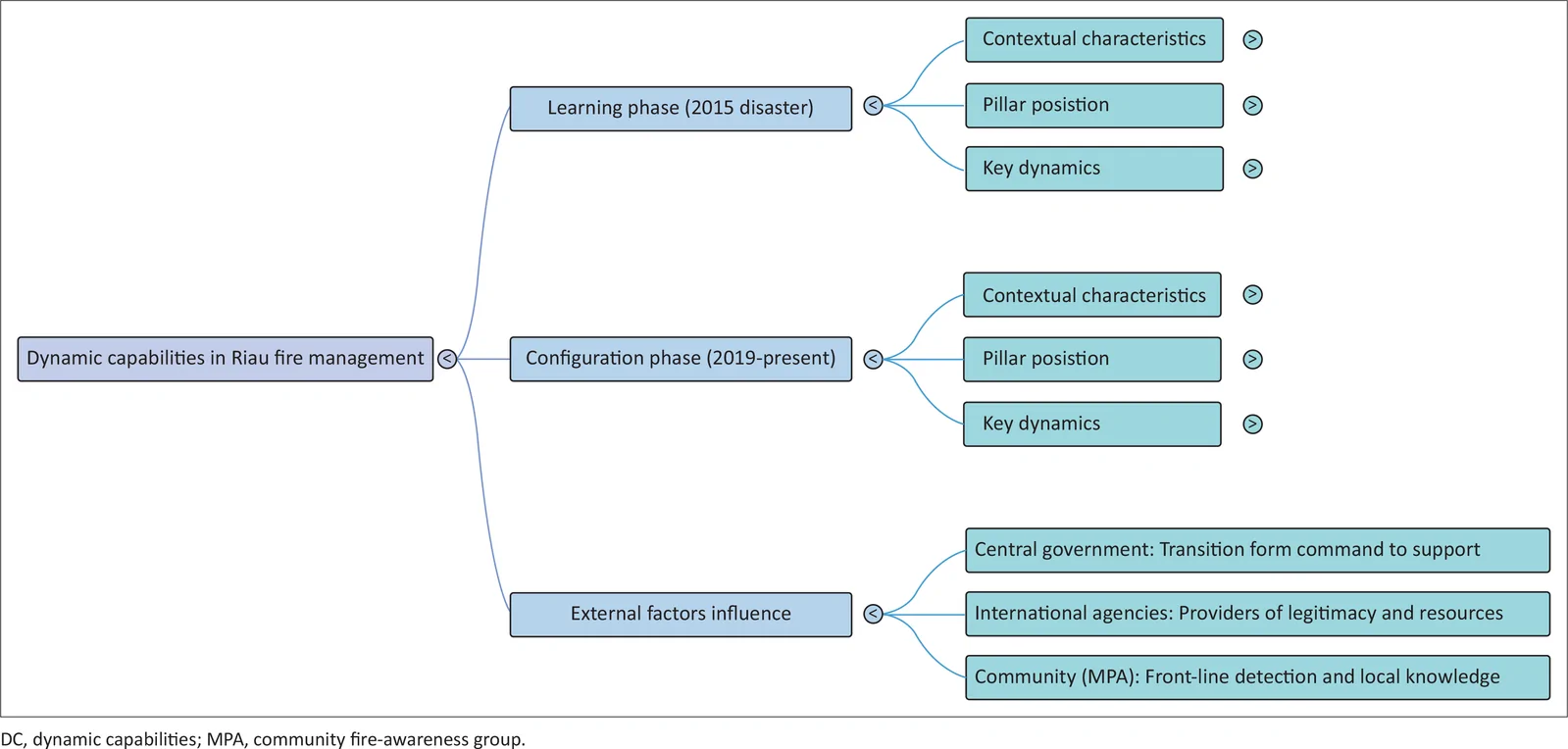
Two phases emerged in dynamic capabilities disaster control with a decentralised system.

The configuration indicates that DC does not occur naturally, it takes internal strength from an organisation to understand what targets it wants to achieve (Ul Akram et al. 2024). This process resembles an evolution, demonstrating the direction of integration, coordination and regulation, which serve as a form of transformation in response to unpredictable conditions. Table 1 provides a conceptual breakdown and includes the practical application of the DC pillars (Teece 2007). Ideal conditions cannot be directly linked to empirical findings in the practice of disaster management decentralisation in Riau. During the 2015 disaster, decentralisation failed as a result of local governments’ lack of preparedness for disaster management, necessitating recentralisation. Changes demonstrating transformation driven by independent initiatives emerged after the 2019 disaster. Figure 1 clearly illustrates the workings of Table 1, representing a key finding of this study. It depicts how the Dynamic Capability (DC) process takes shape within a public organisation, specifically within the scope of local government. Two phases were identified in the development of this capability, revealing that external factors become internalised within the public organisation; this aligns with the evolution of disaster studies, which emphasises the necessity of collaboration between central and local governments.

**TABLE 1:** Operationalisation of dynamic capabilities in local governments.

Pillar	Explanation	Ideal conditions
Sensing	Local governments actively conduct situation analysis and monitor the environment	Able to read data and a measurable design
Seizing	The local government translates the results of the sensing pillar into effective implementation	Establish strategic decisions with measurement
Transform	Local governments conduct performance evaluations and strategy adjustments	Realising long-term adaptive and flexible transformation

These findings validate research indicating that failures in the configuration process can still occur because of negligence within internal resources. According to Zahra’s argument, the cause of this DC failure is the deliberate lack of operational knowledge and the misalignment of leadership interests between the demands of the annual fire alert and other development options (Zahra, Sapienza & Davidsson 2006). Leadership is an important factor in determining which agency to lead, based on the needs and interests at the local government level (Panagiotopoulos, Protogerou & Caloghirou 2023). The absence of DC is equally indicative of the institution’s inability to meet the expected performance targets (Ndrecaj, Tlemsani & Mohamed Hashim 2025). Thus, public organisations that succeed with DC are those that leverage knowledge to continuously adapt and undertake various collaborative approaches (Coulon et al. 2020; Karttunen et al. 2025).

The empirical conditions for the formation of DCs by local governments in response to forest and land fire disasters do not immediately emerge. This necessitates a new phase, namely the construction of Teece’s argument that there is a learning phase before the configuration phase, indicating that DCs have already begun to be formed by local governments. This is the dynamic that arises when implementing disaster decentralisation in regions that are not yet fully prepared. There are differences in the resulting model flow when operationalising this concept in the context of implementing disaster decentralisation in an immature area, such as Riau.

Overall, the findings of this study demonstrate that the development of dynamic capabilities in peatland fire governance in Riau Province did not occur linearly or uniformly, but was shaped through two structurally distinct phases: A learning phase triggered by the 2015 crisis and a configuration phase formed after 2019, each characterised by different contextual features, actor positions and key dynamics. The fundamental distinction between the two phases lies not merely in temporal change, but in a shift in the organising logic of capabilities: From reactive-dependent response towards an asymmetric configuration in which the Provincial Environmental and Forestry Agency advanced through double-loop learning while the Regional Disaster Management Agency experienced stagnation, further distorted by the moral hazard introduced by community-based fire crews (MPA). This pattern cannot be separated from the influence of external factors operating in a layered fashion, the central government transitioning from a command to a support role, international agencies serving as providers of legitimacy and resources, and local communities functioning as frontline detection actors, which collectively shaped the character of a networked dynamic capability. The findings thus affirm that dynamic capabilities in the public sector are not solely dependent on the internal capacity of organisations, but are conditioned by the relational architecture among actors operating within a complex governance ecosystem.

### Disaster management under Indonesia’s decentralisation system: Lessons from Riau

Indonesia’s disaster management system underwent a fundamental structural transformation with the enactment of *Law No. 24 of 2007* on Disaster Management, which formally decentralised disaster governance responsibilities to regional governments. Prior to this legislation, disaster response in Indonesia was characterised by a highly centralised and reactive pattern, in which regional governments functioned primarily as technical executors and extensions of the central government rather than as independent actors with strategic authority. The 1997 peatland fire disaster in Riau illustrates this pre-decentralisation logic clearly: All key decisions, from weather-modification operations conducted by Agency for the Assessment and Application of Technology to aircraft deployment, were initiated and controlled by the central government, while provincial authorities were confined to field-level tasks such as mobilising personnel, distributing masks and supporting firefighting efforts. No strategic space was afforded to the provincial level, and the absence of structured coordination mechanisms meant that crisis management remained reactive, unstructured and entirely top-down.

The shift to decentralisation through *Law No. 24 of 2007* institutionalised regional disaster management by establishing Regional Disaster Management Agencies (BPBD) in every local government. This reform was premised on the logic that regional governments, by virtue of their proximity to affected communities and familiarity with local conditions, are better positioned to lead rapid, contextually appropriate disaster responses. Decentralisation was thus framed not merely as administrative devolution but as a mechanism for building local disaster governance capacity, enabling regional governments to develop their own regulatory frameworks, planning instruments and budget allocations for disaster risk reduction. However, evidence from Riau’s experience across the 1997, 2015 and 2019 crises reveals a persistent structural tension between the formal mandate of decentralisation and the practical dependency of regional governments on central government intervention. Despite the institutional presence of BPBD as the designated leading actor at the regional level, the effectiveness of regional disaster management has remained heavily conditioned by political support, fiscal capacity and logistical transfers from the central government and BNPB, a pattern corroborated by the World Bank’s 2020 assessment identifying institutional fragmentation and weak regional fiscal capacity as the primary obstacles to genuine disaster management autonomy in Indonesia.

The Riau case, spanning nearly three decades of recurring karhutla events, exposes the structural limits of decentralised disaster governance when institutional maturity at the regional level remains uneven and when the anthropogenic drivers of disaster, particularly land governance failures and peatland conversion, are not systematically addressed. The transition from the 1997 crisis, in which no regional institutional capacity existed, to the 2015 crisis, which revealed reactive-dependent governance with failed coordination between BPBD and the Provincial Environmental and Forestry Agency, and subsequently to the 2019 configuration, which demonstrated asymmetric capability development across agencies, reflects not merely the passage of time but the gradual and still incomplete construction of a decentralised disaster governance architecture. External actors, including the central government, international agencies and community-based fire crews (MPA), continued to play structurally significant roles in shaping regional capability trajectories, underscoring that decentralisation in disaster management does not produce autonomous regional capacity automatically but is conditioned by the relational and institutional architecture within which regional governments operate.

### Dynamic capabilities demonstrate commitment to disaster management

Since 1997, Indonesia has progressively reformed its disaster management architecture (Simarmata & Suryandaru 2015), culminating in the 2020 Presidential Instruction that repositioned regional governments as the primary actors in forest and land fire governance. Under this framework, governors serve as provincial fire task force commanders; BPBD is strengthened as the coordinating body at the district and city levels; disaster budgeting is integrated into regional development plans; and both corporate land users and community groups are formally incorporated into prevention and response mechanisms. Government efforts are organised across three stages: Prevention, response and post-fire recovery – encompassing fire patrols, early warning systems, weather modification, peatland restoration, hotspot verification and law enforcement.

However, these mechanisms remain predominantly downstream interventions that address the symptoms of fire rather than its structural causes. Community awareness remains shallow, largely limited to awareness of burning prohibitions rather than understanding of fire as a governable disaster. Meanwhile, the central government’s policy attention has progressively shifted towards climate change as a macro agenda, supported by international funding, while forest and land fire management is increasingly treated as a sub-issue. This institutional and political displacement reveals a critical gap: The architecture for fire governance exists, but its coherence is undermined by fragmented attention, weak upstream accountability and a persistent mismatch between formal mandates and substantive commitment at both central and regional levels.

Building (the process of forming dynamic capabilities) DC for disaster conditions, which also aims to find new capabilities because of performance gaps and crises (Piening 2013). Continuous evaluation of organisational performance is the foundational activity through which new capabilities are built and requires adequate resources to support exploration and problem-solving. In the context of DC, this process does not emerge instantaneously. Building DC requires a prior stage of organisational readiness, establishing trust-based collaboration and supportive leadership, before the three micro-level pillars of sensing, seizing and transforming can operate effectively. This sequence explains why DC development in public organisations is inherently gradual and path-dependent (Pablo et al. 2007).

The formation of dynamic capabilities in disaster governance is therefore not solely an organisational matter it is an actor-dependent process. Organisational leaders must first build a shared understanding of the need for change and innovation, while technical teams serve as the operational backbone, through task forces, translating strategic decisions into field-level action. Beyond internal actors, community-based fire crews (MPA) require specific regulatory frameworks to ensure that every village maintains a first line of defence against fire. Corporate land users present a distinct dynamic: Their behavioural discipline has been driven more by international certification requirements than by domestic regulatory enforcement, exposing a structural accountability gap that demands a clearer definition of corporate responsibility in fire-prone concession areas. Together, these actors form the relational architecture within which dynamic capabilities are or fail to be developed.

Local governments often have limited resources to deal with major disasters and need assistance from the central government (Ishiwatari, Aldrich & Sasaki 2023). During the Indonesian earthquake disaster, local governments, such as those in Bantul, had limitations in terms of human resources, policies and leadership (Kusumasari & Alam 2012). The local government in Brebes also faces coordination problems regarding disaster management (Wahyunengseh & Pamungkas 2025). In the United States, since 2009, collaboration between local governments and various sectors has been integrated into national disaster management policies (Lee & Mossberger 2009). Local governments with new disaster management capabilities are better prepared to reduce the impact of disasters than those with centralised disaster management systems (Tselios 2021).

**FIGURE 2:**
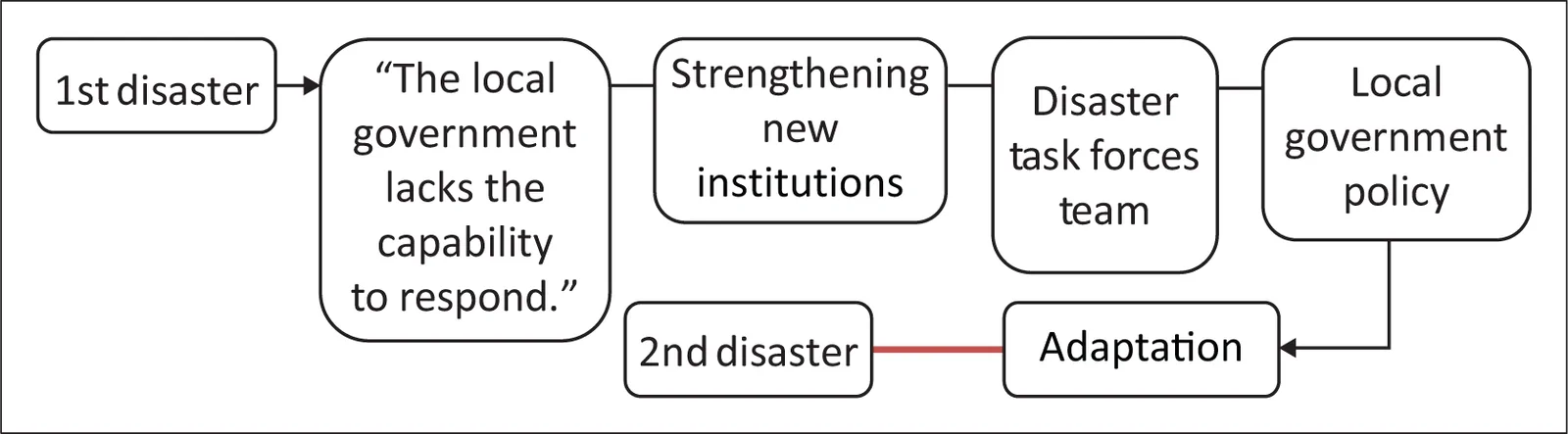
Forest and land fire disaster response patterns in Indonesia (2015–2025).

Figure 2 illustrates the pattern of forest and land fire disaster response in Riau Province between 2015 and 2025, capturing the non-linear trajectory through which dynamic capabilities were gradually constructed. The first disaster (2015) did not immediately produce capability; instead, it produced an institutional recognition of incapacity, which became the critical trigger for a sequence of organisational responses: The strengthening of new institutions, the formation of technical task forces and the consolidation of regional government policy. This sequence reflects the learning phase as an additional construction prior to the emergence of dynamic capabilities, demonstrating that public organisations operating under immature decentralisation cannot directly activate the sensing-seizing-transforming pillars without first resolving foundational institutional deficits. The placement of the second disaster (2019) at the midpoint of the diagram, rather than at the beginning, is analytically significant. It signifies that when the 2019 crisis occurred, the regional government was no longer in a position of complete institutional absence; the prior learning phase had produced a nascent governance architecture. The outcome was therefore no longer incapacity but adaptation represented by the red arrow connecting the second disaster directly to the adaptation node. This adaptation, however, is depicted as an open-ended condition rather than a terminal achievement, reflecting the argument that dynamic capabilities in the public sector are not a fixed endpoint but a continuously renewed process of organisational transformation conditioned by crisis, learning and relational architecture among actors.

A key finding of this study is the regional government’s capacity to strategically seize opportunities emerging from crisis, most concretely demonstrated through the formulation and implementation of the Riau Hijau policy. Far from a declarative slogan, this policy functions as an integrative instrument that embeds climate change considerations into regional development planning documents and budget structures, marking a fundamental reorientation in which environmental governance has shifted from a narrow technical forestry concern to a central pillar of Riau’s broader economic and social development agenda. This reorientation represents the seizing pillar in operation: The regional government identified the post-disaster window as a strategic opportunity to restructure its institutional priorities, translating external pressure from international scrutiny, central government directives and recurring fire crises into enduring policy architecture.

Yet the trajectory of dynamic capability formation in Riau does not conform to Teece’s idealised linear model. What this study reveals instead is an asymmetric-substitutional pattern, in which macro-level advances in international diplomacy, regulatory reform and policy integration coexist with persistent operational deficits at the grassroots level. Hotspot data collected in real-time, for instance, remained largely confined to administrative reporting rather than being systematically deployed as a strategic mitigation tool, exposing a structural gap between institutional ambition and operational execution. This gap is not incidental; it reflects the inherent tension within decentralised disaster governance between the pace of policy innovation at the leadership level and the slower accumulation of technical capacity at the field level. The compensatory causality at work here means that strength in one dimension of macro-level policy and diplomatic positioning partially substitutes for, rather than catalyses, the development of micro-level operational capability.

What has stabilised this otherwise fragile trajectory is leadership continuity with a coherent environmental vision. Successive regional leaders, maintaining a consistent commitment to the Riau Green framework, have served as an institutional anchor, progressively orienting Riau towards a collaborative governance architecture that now extends to engagement with United Nations Environment Programme (UNEP) and Indonesia’s Forest and Other Land Use (FOLU) Net Sink programme. Simultaneously, deliberate steps towards institutional self-sufficiency, most notably the establishment of SIPAKAR in 2021 as an independent fire data management system, signal that the regional government has begun internalising the informational infrastructure necessary for genuine dynamic capability. Taken together, these developments affirm that dynamic capabilities in the public sector are neither a fixed achievement nor a spontaneous organisational quality, but a politically embedded, crisis-driven and continuously renewed process of transformation – one in which the distance between institutional aspiration and operational reality remains the most consequential variable to govern. In this sense, fiscal autonomy, adaptive capacity, public value creation and institutional trust are not parallel variables but mutually reinforcing conditions – and it is precisely their convergence, as demonstrated by the Riau case that determines whether decentralised disaster governance produces genuine regional capability or merely perpetuates dependency under a different institutional label.

## Conclusion

This study advances a central thesis: Dynamic capabilities in decentralised public-sector organisations are not properties that organisations possess but conditions that recurring crises force them to construct; in the context of disaster governance, this construction is never complete. The Riau case demonstrates that when decentralisation precedes institutional maturity, the standard Teece sequence of sensing, seizing and transforming cannot operate as designed. A prior learning phase is required, in which the organisation first confronts its own incapacity, accumulates crisis-driven knowledge, and builds the minimum institutional infrastructure necessary for dynamic capabilities to function. What makes disaster contexts analytically distinctive is precisely this compulsory character: Unlike private sector organisations that may choose when to develop capabilities, public organisations managing recurring disasters have no such discretion; each crisis either accelerates or exposes the limits of whatever capability has been constructed. The theoretical contribution of this study lies in three additional constructions that extend Teece’s framework for public sector disaster governance: A learning phase as a precondition, an asymmetric-substitutional pattern in which macro-level policy advances compensate for rather than catalyse micro-level operational capacity, and networked dynamic capability, in which no single agency’s resilience is separable from its relational ecosystem. For future research, the critical question is whether this pattern is transitional, resolving as institutional maturity deepens or structurally endemic to decentralised disaster governance in developing country contexts.
